# Okadaic Acid: A Tool to Study the Hippo Pathway

**DOI:** 10.3390/md11030896

**Published:** 2013-03-14

**Authors:** Yutaka Hata, Shikshya Timalsina, Sainawaer Maimaiti

**Affiliations:** 1 Department of Medical Biochemistry, Graduate School of Medicine, Tokyo Medical and Dental University, Tokyo 113-8519, Japan; E-Mails: ma120036@tmd.ac.jp (S.T.); sainmbc@tmd.ac.jp (S.M.); 2 Department of Psychotherapy, The Fourth People’s Hospital of Urumqi, Urumqi 830000, China

**Keywords:** Hippo pathway, kinase, okadaic acid, phosphatase

## Abstract

Mammalian Ste20-like kinases 1 and 2 (MST1 and MST2) are activated in NIH3T3 cells exposed to okadaic acid. The Hippo pathway is a newly emerging signaling that functions as a tumor suppressor. MST1 and MST2 work as core kinases of the Hippo pathway and their activities depend on the autophosphorylation, which is negatively regulated by protein phosphatase 2A (PP2A). Okadaic acid has been frequently used to enhance the phosphorylation of MST1 and MST2 and to trigger the activation of the Hippo pathway. However other components of the Hippo pathway could also be targets of okadaic acid. In this review we first briefly summarize the molecular architecture of the Hippo pathway for the reference of researchers outside the field. We explain how MST kinases are regulated by PP2A and how okadaic acid activates MST2. Thereafter we discuss which components of the Hippo pathway are candidate substrates of protein phosphatases and which points we need to consider in the usage of okadaic acid to study the Hippo pathway.

## 1. Introduction

The Hippo pathway is a newly emerging tumor suppressor signaling [1,2]. It was originally identified in *Drosophila* as the signaling that regulates organ size. The pathway is well conserved in mammals. As the dysfunction of the pathway is frequently observed in human cancers and results in poor prognosis, it has been studied as a tumor suppressor signaling in the cancer research field. Recent studies have also revealed that the Hippo pathway is important in the regulation of tissue stem cells [3,4]. The Hippo pathway has come to attract attentions in the field of regeneration medicine. Mammalian Ste20-like kinases 1 and 2 (MST1 and MST2) are core components of the Hippo pathway. MST1 and MST2 were identified as kinases that were activated in NIH3T3 cells by the treatment of okadaic acid [5]. Okadaic acid inhibits protein phosphatase 1 (PP1) and protein phosphatase 2A (PP2A), which negatively regulate MST1 and MST2. Accordingly okadaic acid has been widely used to experimentally activate the Hippo pathway. However PP1 and PP2A target not only MST1 and MST2 but also other components of the Hippo pathway. Moreover it is necessary to consider that PP1 and PP2A have a wide range of substrates, which are irrelevant of the Hippo pathway, and that okadaic acid may influence other phosphatases than PP1 and PP2A. In this brief review we first introduce the basal architecture of the mammalian Hippo pathway and describe which components are potential substrates of PP1 and PP2A. We will discuss the potential pitfalls in using okadaic acid as the activator of the Hippo pathway. 

## 2. The Mammalian Hippo Pathway

**Figure 1:**
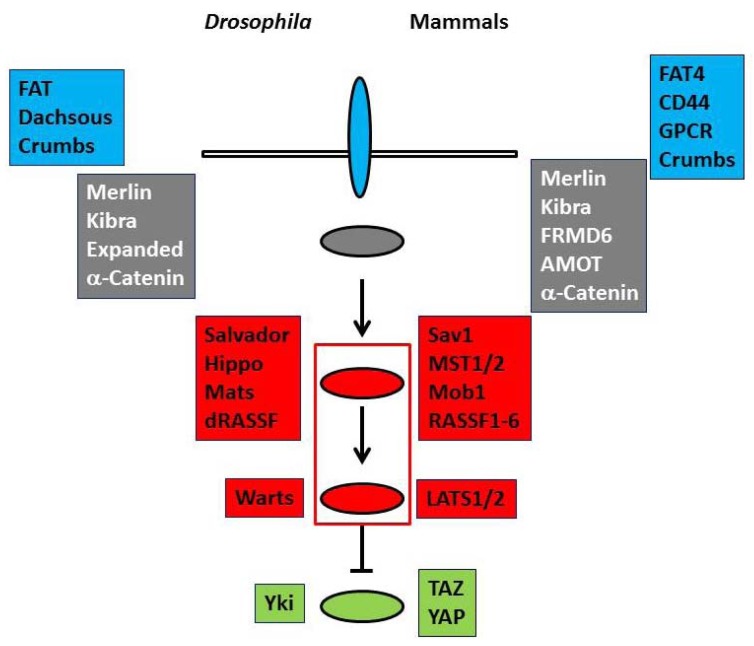
The basal molecular architecture of the Hippo pathway. The components of *Drosophila* (left) and mammalian (right) Hippo pathway are summarized. 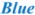 and 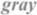 panels and ovals represent membrane proteins and membrane-associated proteins of the upstream regulators, respectively. 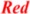 and 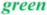 panels and ovals show core components that include serine/threonine kinases and transcriptional co-activators that are phosphorylated and negatively regulated by kinases. Please refer to text for details.

*Drosophila* Hippo pathway has two serine/threonine kinases (Hippo and Warts) and their regulators (Salvador, Mats, and dRASSF) as the core components. Cell membrane proteins (FAT, Dachsous, and Crb) and membrane-associated proteins (Merlin, Kibra, Expanded, and α-catenin) are regarded as upstream regulators [1,2] (Figure 1, left). When the pathway is activated, Hippo phosphorylates and activates Warts, which in turn phosphorylates a transcriptional co-activator, Yorkie (Yki), and negatively regulates it [6]. Yki co-works with a transcriptional factor, Scalloped to up-regulate cell cycle promoting and anti-apoptotic gene transcriptions. Therefore the dysfunction of the Hippo pathway results in the hyperactivity of Yki and leads to organ hypergrowth and tumorigenesis. 

The basal molecular structure of the pathway is conserved in mammals (Figure 1, right). Merlin and Kibra function as upstream regulators. FAT4 and FRMD6 (Willin) are proposed to be homologs of FAT and Expanded, respectively. Mammals have CD44, G protein-coupled receptors and AMOT family proteins as additional upstream regulators [1,2,7,8,9]. In mammals the core components comprise two Hippo homologs (MST1 and MST2), two Warts homologs (large tumor suppressor kinases (LATS1 and LATS2)), Salvador homolog (Sav1), multiple Mats homologs (Mobs), and six dRASSF homologs (RASSF1 to RASSF6). LATS kinases phosphorylate and inhibit two transcriptional co-activators, Transcriptional co-activator with PDZ-binding motif (TAZ) and Yes-associated protein (YAP). In many cells, the pathway is inactive at the low cell density, but is activated when cells reach confluence or are exposed to stress, suggesting that the pathway is implicated in contact inhibition and check point. The mutations and the suppressed expressions by DNA hypermethylation of the components are frequently observed in human cancers. These findings support that the Hippo pathway works as the tumor suppressor signals. Furthermore TAZ and YAP co-work with various transcriptional factors and play important roles to regulate stem cells. Therefore Hippo pathway is involved in various organogenesis, tissue differentiation, and tissue regeneration in the cell and tissue context-dependent manner [3,4,6].

## 3. Regulation of MST1/2 Kinase Activities by Okadaic Acids

MST1 and MST2 were reported as kinases responsive to stress a long time before the discovery of the Hippo pathway [5]. NIH3T3 cells were treated with okadaic acid and activated kinases were identified by a myelin basic protein in-gel assay. The kinases turned out to be related to yeast Ste20 kinase. Thereafter okadaic acid has been used as an experimental reagent to activate MST kinases and the Hippo pathway.

**Figure 2:**
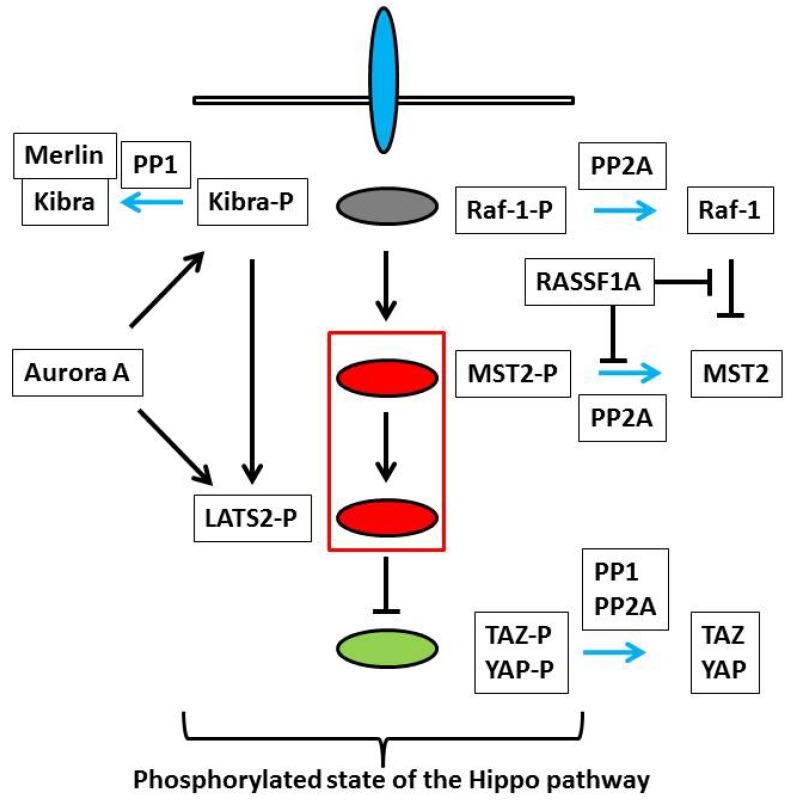
Potential targets of okadaic acid in the Hippo pathway. When the Hippo pathway is active, MST2, TAZ, and YAP are phosphorylated. Okadaic acid inhibits PP1 and PP2A and enhances these phosphorylations. Raf-1 negatively regulates MST2. RASSF1A antagonizes the interaction between Raf-1 and MST2, and blocks the dephosphorylation of MST2. Okadaic acid also enhances the inhibitory phosphorylation of Raf-1 and releases MST2 from the negative regulation by Raf-1. Kibra is phosphorylated by Aurora A and is dephosphorylated by PP1. Kibra promotes the Aurora A-mediated phosphorylation of LATS2. The biological significance of the Aurora A-mediated phosphorylation of Kibra in the Hippo pathway is not yet clear, but the phosphorylation affects the interaction between Kibra and Merlin. 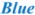, 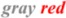 and 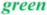 ovals show the upstream regulators, the core components, and the transcriptional co-activators as in Figure 1. 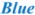 arrows represent the dephosphorylation of various proteins, which are blocked by okadaic acid. Other components of the Hippo pathway such as Mob1 and LATS kinases can also be targets of okadaic acid.

MST1 and MST2 are highly homologous in the kinase domain but diverged in the *C*-terminal non-kinase domain [10,11,12]. MST1 is activated by the cleavage of the inhibitory domain, while the cleavage of MST2 is not remarkable. Autophosphorylation is essential for the kinase activity of full MST kinases. The dimerization facilitates the autophosphorylation. Under serum deprivation Raf-1 interferes with MST2 dimerization and recruits PP2A to dephosphorylate MST2 [13]. Conversely MST2 maintains PP2A expression level and PP2A dephosphorylates the inhibitory phosphorylation at Serine 259 of Raf-1 to maintain the activity of Raf-1 [14]. Overall the inhibition of PP2A by okadaic acid results in the suppression of the inhibitory effect of Raf-1 on MST2. In *Drosophila* the affinity purification followed by mass spectrometry analysis and genetic studies identified PP2A complex as a negative regulator of the Hippo pathway [15]. dRASSF promotes the association of PP2A complex to Hippo and inactivates it. In contrast mammalian RASSF1A releases MST kinases from Raf-1 and counteracts the dephosphorylation of MST kinases to activate them [16,17]. These reports are apparently inconsistent and suggest that *Drosophila* RASSF and mammalian RASSF work in distinct manners. However, in any case, okadaic acid, which inhibits PP1 and PP2A, up-regulates MST activities (Figure 2). Therefore it is rational to use okadaic acid as an activator of the Hippo pathway. 

## 4. TAZ and YAP as Substrates of PP1 and PP2A

TAZ and YAP are encoded by different genes, but have similar molecular structures [6]. Unphosphorylated TAZ and YAP remain in the nucleus and induce gene transcriptions. When the Hippo pathway is activated, TAZ and YAP are phosphorylated by LATS kinases and recruited from the nucleus to the cytoplasm. The phosphorylation also induces degradation of TAZ and YAP. Proteomic analyses by several research groups revealed the association of PP1 and PP2A with TAZ and YAP [18,19,20]. PP1 and PP2A dephosphorylate TAZ and YAP. From this point of view okadaic acid directly inhibits the activities of TAZ and YAP by maintaining the phosphorylation (Figure 2).

## 5. Other Candidate Substrates of PP1 and PP2A in the Hippo Pathway

As described above okadaic acid can activate the Hippo pathway at the levels of MST kinases, TAZ and YAP. To be more complicated, many components of the Hippo pathway show robust phosphorylation. For instance, Sav1, Mob1, and RASSF6 are phosphorylated by MST kinases at multiple sites. Mob1 functions as a scaffold to link MST kinases to LATS kinases and to facilitate the MST kinase-mediated phosphorylation of LATS kinases [21,22,23]. Mob1 also directly activates LATS kinases. Both of the scaffolding function and the direct activation are enhanced by phosphorylation of Mob1. LATS kinases activities depend on phosphorylation by MST kinases. The molecular mechanism underlying the dephosphorylation of Mob1 and LATS kinases is not yet clarified. Okadaic acid enhances the phosphorylations of Mob1 and LATS kinases [17,24]. Although the enhancement can be the secondary effect of the activation of MST kinases, PP1 and PP2A may directly affect Mob1 and LATS kinases. In such cases okadaic acid should modulate the Hippo pathway through the control of the phosphorylation states of these molecules. More specifically okadaic acid is likely to influence Kibra, an upstream regulator of the Hippo pathway. Kibra is phosphorylated by Aurora A at Serine 539. Kibra associates with LATS kinases and stabilizes them. Kibra also promotes Aurora A-mediated phosphorylation of LATS kinases. Serine 589 phosphorylation of Kibra has no effect on YAP phosphorylation by LATS kinases but is likely to stimulate the dissociation of Kibra from Merlin in the nocodazole-treated cells. [25] (Figure 2). Although the impact of the interaction between Kibra and Merlin on the Hippo pathway is not clear, okadaic acid treatment may influence the Hippo pathway through the regulation of Kibra. 

## 6. Conclusions

In almost all studies researchers use 1 μM okadaic acid to activate the Hippo pathway and induce apoptosis [26,27]. When the Hippo pathway is active, most components are in phosphorylated forms (Figure 2). Hence okadaic acid can be trusted to activate the Hippo pathway. However, several potential pitfalls need to be considered. First okadaic acid is a potent inhibitor for PP1 (IC_50_ 20 nM) and PP2A (IC_50_ 0.2 nM) but it also inhibits PP4, PP5, and PP6. More importantly, even supposing okadaic acid only inhibits PP1 and PP2A, these phosphatases have a wide variety of substrates. Okadaic acid treatment results in various cellular events irrelevant of the Hippo pathway. Consistently okadaic acid-induced apoptosis can be attenuated by silencing of MST kinases but only partially in most cases, which signifies that other pathways also contribute to okadaic acid-induced apoptosis. In conclusion, to precisely dissect the Hippo pathway, it is essential to develop more specific activators than okadaic acid.
